# COVID-19 Impact on Pathology Education Program in Iran

**DOI:** 10.30699/ijp.2024.2004823.3130

**Published:** 2024-07-24

**Authors:** Shabnam Mashhadi, Alireza Abdollahi, Fereshteh Ameli, Fatemeh Nili

**Affiliations:** Department of Pathology, Imam Khomeini Cancer Institute, Tehran University of Medical Sciences, Tehran, Iran

**Keywords:** COVID-19, Medical residents, Online education, Pathology

## Abstract

**Background & Objective:**

The coronavirus disease brought worldwide uncertainty, and Iran was affected by it as well as many other countries in the world. Halting face-to-face education due to social distancing and resident re-employment in clinical wards leads to defective education. The aim of this survey was to evaluate the advantages and disadvantages of modifications made in pathology residency education in Iran.

**Methods:**

This online survey was conducted on all pathology residents in Iran. An online 30-item questionnaire was developed and used in this study.

**Results:**

Sixty residents (88.3% female) participated in this survey. The majority (70%) of the residents were over 30 years old. Fifty percent of the responders reported that their personal life was influenced by the pandemic. Skyroom and Adobe Connect were the most common platforms for online education with overall satisfaction of 65%. The webinars were considered suitable by 51.7% of the responders. Concerns at work were reported by 48.3% of the residents, while 78.3% reported being exposed to the disease and 55% reported being infected. Concerns about transmission of the disease to family members were reported by 90% of the responders.

**Conclusion:**

This study showed that the pathology residency modifications were successful in providing education. However, their social and educational life characteristics might affect their satisfaction with online education.

## Introduction

The first cases of the novel respiratory disease caused by the severe acute respiratory syndrome Coronavirus 2 (SARS Cov 2) were reported in December 2019, which led to the naming of the disease coronavirus disease 2019 (COVID-19) (1). The disease spread rapidly worldwide bringing both fears and uncertainties and Iran was no exception. Self-care regulations were implemented by Iran's national health system to limit the disease spread (2). As social distancing and lockdown took place, actions needed to be taken for both the private and social lives of individuals. The closure of schools and educational institutions and reduced working hours led to an insufficient educational system (3). The healthcare workers’ routine tasks were altered by the rush of infected patients to hospitals and health centers. Various hospital wards as well as intensive care units were forced to admit COVID-19 patients resulting in the cancelation of elective surgeries. Moreover, physicians and medical staff were supposed to maintain the front line regardless of their subspecialty (3). 

In terms of medical education, the residency training program was severely affected by the COVID-19 pandemic. The main parts of residency education include clinic and routine medical practice, both of which were affected due to the closure of outpatient clinics and the cancellation of elective procedures. Even limited operations, mainly emergency procedures, were also affected by COVID-19 due to the complexities in the management of patients with COVID-19. On the other hand, clinics were occupied with infected patients, which had a great laboratory and radiologic burden. The exponential surge in requested laboratory tests on the one hand and social distancing on the other, impaired laboratory students' education. Another constraint for residency education was the obligatory shifts in COVID-19 wards due to patient overload and emergency staff burnout.

Pathology residents need to be prepared for both clinical and surgical issues. Therefore, pathology residency education also required major modifications during the COVID-19 pandemic. One of these modifications was the use of virtual education. Besides, as pathology residents were obliged to be present at the front line of COVID-19 patient care, these new tasks required urgent preparation and taking shifts in the emergency and COVID-19 wards and thus, further restricted the active hours of residency education. This study was designed to estimate the pros and cons of the renewed pathology educational system in Iran during the COVID-19 pandemic.

## Material and Methods

An online survey was conducted during the COVID-19 pandemic in 2022 in Iran. The study questionnaire was prepared by two attending physicians. The questionnaire included 30 multiple-choice questions in four domains. The questionnaire domains included demographic data, educational life, personal life, and social life. 

The questionnaire was uploaded to a private Google Forums sheet. The study announcement was sent via instant message to all Iranian pathology residents by the Iranian Society of Pathology. The link to the online questionnaire was placed in the message. By clicking on the link, residents were directed to the form. The first page of the form included information about the objectives of the study and residents who were willing to participate in the survey were asked to click on the agree hyperlink as online consent. Only after giving consent, residents could access the questionnaire. The form was designed in a way that each pathologist could respond only once.

**Statistical Analysis**

Data was analyzed using SPSS version 23 (SPSS Inc., Chicago, Ill., USA). Descriptive statistics were performed using mean, standard deviation, frequency, and percentage. 

## Results

**Demographic**

Sixty volunteers responded to the questionnaire, among whom 88.3% were female. The majority of the responders (70%) were over 30 years old and were trained at the Tehran University of Medical Sciences (36.7%) followed by Shahid Beheshti University of Medical Sciences (15%). The least participation was observed in three medical universities namely, Mashhad, Kerman, and Yazd. 

Regarding trainees' years of education, 28.3% were in the second year, followed by fourth year (26.7%), third year (18.3%), first year (15%), and graduated residency (11.7%). The participants’ responses to the questionnaire items are presented in Table 1.

**Table 1:** Description of the responders’ answers to the questionnaire items

Question	Response n (%)				
	Completely disagree	Disagree	Abstainer	Agree	Completely agree
Impaired grossing teaching	7 (11.7%)	22 (36.7%)	3 (5%)	18 (30%)	10 (16.7%)
Impaired microscopic teaching	7 (11.7%)	19 (31.7%)	4 (6.7%)	18 (30%)	12 (20%)
Impaired cytology teaching	7 (11.7%)	15 (25%)	7 (11.7%)	19 (31.7%)	12 (20%)
Complex specimens and learning challenge	3 (5%)	12 (20%)	11 (18.3%)	28 (46.7%)	6 (10%)
Impaired clinical teaching	3 (5%)	11 (18.3%)	9 (15%)	23 (38.3%)	14 (23.3%)
Increased time in research	7 (11.7%)	17 (28.3%)	21 (35%)	14 (23.3%	1 (1.7%)
Increased time for self-learning	8 (13.3%)	18 (30%)	7 (11.7%)	21 (35%)	6 (10%)
Low-quality examination	1 (1.7%)	12 (20%)	10 (16.7%)	24 (40%)	13 (21.7%)
Positive impression of virtual learning	2 (3.3%)	9 (15%)	7 (11.7%)	30 (50%)	12 (20%)
Troubles in participating in virtual learning	4 (6.7%)	12 (20%)	6 (10%)	31 (51.7%)	7 (11.7%)

**Educational Life**

According to the questionnaire, 36.7% of responders reported that the Navid offline educational system was useful in learning. Fifty percent of the responders reported that their personal life was influenced by COVID-19, whereas 28.3% reported that their personal lives were not affected by the pandemic. 

The most commonly used virtual platforms were Skyroom and Adobe Connect (Table 2), which showed 65% overall satisfaction. Overall, educational webinars quality was considered good by 34%, and medium by 35% of the responders. The webinar’s frequency was commonly once per month (reported by 41.7% of the responders). By contrast, 25% of the responders reported that the webinars were seldom held. In the case of these webinars’ educational quality, 51.7% were suitable following 33.3% of medium quality.

**Social Life**

Concerns at work were present in 48.3% of the responders. Workplaces provided self-protective items to 58.3% of the responders, although the majority of the responders (78.3%) reported that they were exposed to infected patients. COVID-19-specific diagnostic tests were available for 73.3% of the responders. The positive PCR test was reported in 43.3% of the responders. Fifty-five percent of the responders reported that they were given five-day leave in case of being infected. Pathology residents were responsible for disease management in COVID-19 care units, nonetheless, 11.7% reported that they received training for COVID-19 management. 

**Table 2:** Descriptive of the online platform usage by the responders

Virtual platforms	Frequency	Percent
Sky room	24	40
Big blue button	5	8.3
Adobe Connect	26	43.3
Skype	3	5
Zoom	1	1.7
Total	59	98.3
Missing*	1	1.7

*Universities without online courses

**Personal Life**

One-quarter of the responders reported living with their spouses, while 15% were living alone 3.3% reported having children, and the majority of the responders were single (56.7%). About 23.3% of the responders shared their home with a parent or relative over 60 years of age. More than one-third of the responders (38.3%) reported that their time spent with their family members did not change during the pandemic, while 16.7% of the responders reported that spent more time with their family. Ninety percent of the responders reported struggling with worries about their families’ health status, who had not been infected. The majority of the responders (60%) lived far from their family members. Coronavirus infection burdened anxiety in 71.6% of the responders and 76.7% of the responders felt hopeless regarding their unclear future considering the education they received. 

## Discussion

The COVID-19 pandemic provided the opportunity for modern education technologies, including virtual learning tools, and online courses, to replace face-to-face teaching and self-learning strategy. However, interpersonal collaboration was affected by the pandemic. In this regard, digital pathology was introduced to face this problem (4).

Different countries responded differently to the education restrictions caused by the pandemic. Developed countries, including Canada, employed digital slide learning in anatomical pathology practice (5). In the same fashion Mayo Clinic in the United States utilized online live interactive conferences (6). In developing countries like Iraq, the use of online platforms significantly increased during the pandemic (7). In Turkey, real-time pathology microscopic lectures were held (8).

**Educational Life**

Our survey showed an impressive work of online classes on residential education as evidenced by more than 80% satisfaction with education quality (51.7% suitable and 33.3% medium quality). This finding was in line with the findings of a systematic review of previous studies that indicated 57% satisfaction with online classes among medical students during the COVID-19 pandemic (9). 

The most desirable ongoing hours were morning classes; however, residents faced an increased workload at that time. Skyroom and Adobe Connect meeting rooms were the most used and satisfied residents in both video and audio quality. More than one-third of the respondents were satisfied with offline courses and 25% of the residents reported that the webinars were not held. Difficulties in online participation of the responders might be due to network problems. High internet speed and optimal bandwidth are in demand for online learning (10). Additionally, residents' participation in online courses could have been influenced by their in-hospital practice. 

**Social Life**

Our survey indicated that nearly half of the respondents were worried about the disease, while 58% of the respondents were provided with self-protective items. Based on the findings of a previous study, the most important crisis-related need that could affect the psychiatric health of medical residents in New York City during the COVID-19 pandemic was access to self-protective items. Therefore, it can be hypothesized that low access to these items could have resulted in increased worries among the residents in our study (11). Similarly, a high infection rate based on PCR tests (73.3%), insufficient leave due to heavy workload, and not receiving proper education on COVID-19 management may also be considered as other reasons for the increased worries of the respondents. A systematic review of previous studies has also shown that increased COVID-19 management duties and infection may increase psychological disorders including depression and stress, which could be a cause for the observed high level of worry among the respondents in our survey (12).

**Personal Life**

Our survey showed that a quarter of the responders reported living with their spouses and 3.3% reported having children. Less than one-third of the respondents had a parent or a spouse over 60 years of age. Although a small number of residents lived with vulnerable parents, the majority of the residents were worried about their families’ health status and reported anxiety and hopelessness regarding their future. Similar concerns have been reported by medical students and medical education planners during the COVID-19 pandemic (13). Unknown quality of education and limited clinical practices due to COVID-19 restrictions and increased workload in emergency units may affect the performance of the residents in their future clinical practice (13, 14). More than one-third of the responders (38.3%) reported that their time spent with their family members did not change during the pandemic. In a study conducted on university students in Jordan, 26.3% of the respondents reported that they did not mind restricting their visits to siblings and parents to prevent disease transfer, which was in line with the findings of our survey (15).

In summary, our survey uncovered a more serious impairment in clinical pathology education. Despite social distancing as well as a laboratory workforce, some laboratory divisions were shut down to adjust to the repetitive requested blood tests. Thus, the number of on-duty residents dropped in laboratories and also switched their teaching to E-classes. Another factor that may have affected our residential learning was their stress load. Most of the responders were living with either a spouse or parent, for whom they had mental occupation and worries in terms of disease transmission. Some residents lived separately from their families, which caused difficulties in caring for their families due to COVID-19 safety regulations. To reduce this unwanted pressure, all hospitals provided all available self-protection tools. Also, polymerase chain reaction (PCR) tests were readily available in most of the countries. Surprisingly, nearly half of the residents became infected by the virus. Even though pathology practice is not associated with direct patient contact. This might be because some medical universities utilized pathology residents in COVID-19 care units to expand their disease management capacity. However, most of the pathology residents did not receive proper education for patient triage or treatment. This unspontaneous participation also has added psychological pressure on residents and thus, had a negative effect on their learning. 

This study had some limitations. The responders in our survey were mainly females. Studies have shown that female students benefited more from virtual classes compared to males. Therefore, this unequal participation might make it hard to generalize the findings of this study to the whole population of pathology residents. Additionally, lower stress levels were found to be correlated with better online learning. (16) Therefore, the diversity in the education and social life of the residents could be considered as the main mediators of the effectiveness of medical education. Regarding the diversity of these factors and the study sample size, the statistical adjustment of these variables was not possible in this study. Therefore, it is recommended that further studies be conducted using stratified sampling to achieve equal samples of the participants. Another limitation could be recall bias due to relying on self-report. Therefore, it is recommended that further studies include residents’ academic achievements in the evaluation of the efficacy of online education. 

## Conclusion

The COVID-19 pandemic modified the residency education system. Some of these changes are expected to remain even after the pandemic. Video conferences will still be useful and recorded videos could be offered as offline educational materials. Stress management and providing mental and physical health tools will reduce trainees' stress resulting in better learning.

## References

[1] Zhu N, Zhang D, Wang W, Li X, Yang B, Song J (2020). A novel coronavirus from patients with pneumonia in China, 2019. N Engl J Med.

[2] Sintema EJ (2020). Effect of COVID-19 on the performance of grade 12 students: Implications for STEM education. Eurasia J Mathemat Sci Technol Educat.

[3] Chertoff JD, Zarzour JG, Morgan DE, Lewis PJ, Canon CL, Harvey JA (2020). The early influence and effects of the coronavirus disease 2019 (COVID-19) pandemic on resident education and adaptations. J Am Coll Radiol.

[4] Azimi Khatibani S, Tabatabai S (2021). Virtual Pathology Education in Pandemic Times: Challenges, Solutions and Future Implications. Interdiscip J Virtual Learn Med Sci.

[5] Hojilla C, Armstrong S, Pun C, Hickey TBM, Mete O, Han R (2021). A holistic approach to pathology education during the coronavirus disease 2019 (COVID-19) pandemic. Arch Pathol Lab Med.

[6] Patel R, Hoppman NL, Gosse CM, Hagen-Moe DJ, Dunemann SK, Kreuter JD (2021). Laboratory medicine and pathology education during the COVID-19 pandemic- lessons learned. Acad Pathol.

[7] Hasan DO, Aladdin AM, Amin AAH, Rashid TA, Ali YH, Al-Bahri M (2023). Perspectives on the Impact of E-Learning Pre-and Post-COVID-19 Pandemic-The Case of the Kurdistan Region of Iraq. Sustainability.

[8] Sensu S, Teke O, Demirci M, Kutlu S, Genc BN, Ayalti S (2020). Telepathology in medical education: integration of digital microscopy in distance pathology education during COVID-19 pandemic. Microscopy.

[9] Ahmed H, Mohammed O, Mohammed L, Mohamed Salih D, Ahmed M, Masaod R (2022). Prevalence of medical students? satisfaction with online education during COVID-19 pandemic: A systematic review and meta-analysis [version 2; peer review: 2 approved with reservations, 1 not approved]. MedEdPublish.

[10] Koch LK, Correll-Buss A, Chang OH (2022). Implementation and effectiveness of a completely virtual pathology rotation for visiting medical students. Am J Clin Pathol.

[11] Kaplan CA, Chan CC, Feingold JH, Kaye-Kauderer H, Pietrzak RH, Peccoralo L (2021). Psychological Consequences Among Residents and Fellows During the COVID-19 Pandemic in New York City: Implications for Targeted Interventions. Acad Med.

[12] Gupta P, B K A, Ramakrishna K (2021). Prevalence of Depression and Anxiety Among Medical Students and House Staff During the COVID-19 Health-Care Crisis. Acad Psychiatry.

[13] Daroedono E, Siagian FE, Alfarabi M, Cing JM, Arodes ES, Sirait RH (2020). The impact of COVID-19 on medical education: our students perception on the practice of long distance learning. Int J Commun Med Public Health.

[14] Hunzeker AE, Lang KG, Gao RR, Smith TG, Quinones PJ, Garda AE (2023). Maintaining High Quality Dosimetry Education in Radiation Oncology Medical Residency Amid and Post-COVID-19 Pandemic: A Reimagined Training Rotation. Int J Radiat Oncol Biol Phys.

[15] Almomani EY, Qablan AM, Atrooz FY, Almomany AM, Hajjo RM, Almomani HY (2021). The influence of coronavirus diseases 2019 (COVID-19) pandemic and the quarantine practices on university students' beliefs about the online learning experience in Jordan. Front Public Health.

[16] Nikas IP, Lamnisos D, Meletiou‐Mavrotheris M, Themistocleous SC, Pieridi C, Mytilinaios DG (2022). Shift to emergency remote preclinical medical education amidst the Covid‐19 pandemic: A single‐institution study. Anat Sci Educ.

